# Implementation of Client-Centered Care Coordination for HIV Prevention with Black Men Who Have Sex with Men: Activities, Personnel Costs, and Outcomes—HPTN 073

**DOI:** 10.1007/s40615-021-01209-y

**Published:** 2022-01-08

**Authors:** Darren L. Whitfield, LaRon E. Nelson, Arnošt Komárek, DeAnne Turner, Zhao Ni, Donte T. Boyd, Tamara Taggart, S. Raquel Ramos, Leo Wilton, Geetha G. Beauchamp, Lisa Hightow-Weidman, Steven J. Shoptaw, Manya Magnus, Kenneth H. Mayer, Sheldon D. Fields, Darrell P. Wheeler, for the H. I. V. Prevention Trials Network (HPTN) 073 Study Team

**Affiliations:** 1 School of Social Work, University of Maryland Baltimore, Baltimore, PA USA; 2 School of Nursing, Yale University, New Haven, CT USA; 3 MAP Centre for Urban Health Solutions, Li Ka Shing Knowledge Institute, Unity Health Toronto-St. Michael’s Hospital, Toronto, ON Canada; 4 Department of Probability and Mathematical Statistics, Faculty of Mathematics and Physics, Charles University, Prague, Czech Republic; 5 College of Nursing, University of South Florida, Tampa, FL USA; 6 School of Medicine, Yale University, New Haven, CT USA; 7 College of Social Work, The Ohio State University, Columbus, OH USA; 8 Department of Prevention and Community Health, Milken Institute School of Public Health, George Washington University, Washington District of Columbia, USA; 9 Department of Social and Behavioral Sciences, Yale School of Public Health, New Haven, CT USA; 10 Department of Human Development, State University of New York at Binghamton, Binghamton, NY USA; 11 Faculty of Humanities, University of Johannesburg, Johannesburg, South Africa; 12 Statistical Center for HIV/AIDS Research & Prevention, Fred Hutchinson Cancer Research Center, Seattle, WA USA; 13 Division of Infectious Diseases, School of Medicine, University of North Carolina Chapel Hill, Chapel Hill, NC USA; 14 Division of Family Medicine, David Geffen School of Medicine, University of California Los Angeles, Los Angeles, CA USA; 15 Department of Epidemiology, Milken Institute School of Public Health, George Washington University, Washington District of Columbia, USA; 16 The Fenway Institute, Fenway Health, Boston, MA USA; 17 Ross and Carol Nese College of Nursing, The Pennsylvania State University, University Park, PA USA; 18 Iona College, New Rochelle, NY USA

**Keywords:** Client-centered, Care coordination, Black MSM, Pre-exposure prophylaxis, HIV prevention

## Abstract

**Background:**

Black men who have sex with men (MSM) experience disproportionate rates of HIV infection in the USA, despite being no more likely to engage in sexual risk behaviors than other MSM racial/ethnic groups. HIV pre-exposure prophylaxis (PrEP) has been shown to reduce risk of HIV acquisition; however, rates of PrEP use among Black MSM remain low. Clinical, psychosocial, and structural factors have been shown to impact PrEP use and adherence among Black MSM. Care coordination of HIV prevention services has the potential to improve PrEP use and adherence for Black MSM, as it has been shown to improve HIV-related care outcomes among people living with HIV.

**Methods:**

Client-centered care coordination (C4) is a multi-level intervention designed to address clinical, psychosocial, and structural barriers to HIV prevention services for Black MSM within HPTN 073, a PrEP demonstration project among Black MSM in three cities in the USA. The current study examined the implementation process of C4, specifically investigating the activities, cost, time, and outcomes associated with the C4 intervention.

**Results:**

On average, participants engaged in five care coordination encounters. The vast majority of care coordination activities were conducted by counselors, averaging 30 min per encounter. The cost of care coordination was relatively low with a mean cost of $8.70 per client encounter.

**Conclusion:**

Although client-centered care coordination was initially implemented in well-resourced communities with robust HIV research and service infrastructure, our findings suggest that C4 can be successfully implemented in resource constrained communities.

## Background

Black men who have sex with men (MSM) remain disproportionately affected by the HIV epidemic. In 2019, Black MSM comprised more than one-third of new HIV infections in the USA [1]. HIV pre-exposure prophylaxis (PrEP) is a biomedical HIV prevention option that is underutilized among Black MSM. Daily oral PrEP has shown success in reducing the sexual transmission of HIV in several large-scale randomized control trials among MSM, with effectiveness estimates exceeding 90% [2]; however, PrEP use remains low for Black MSM in the USA. The current available evidence suggests that a substantial proportion of Black MSM are not using PrEP, stop using PrEP soon after initiating, or do not consistently adhere to PrEP [3, 4].

Clinical, psychosocial, and structural factors are associated with PrEP use and adherence among Black MSM. In prior studies, Black MSM with a history of previous STIs, substance use, and high perceived HIV risk are more likely to use PrEP [5–7], while those with depressive symptoms and experienced PrEP-related stigma are less likely to use PrEP [8]. Furthermore, structural factors such as living in poverty, lack of health insurance, inadequate healthcare access, and provider bias contribute to decreased PrEP use among Black MSM [7, 9, 10].

Care coordination has been shown to improve adherence to anti-retroviral (ARV) medications among people living with HIV, but few studies have been conducted that have implemented care coordination to support adherence to ARVs for PrEP [11–13]. Care coordination is a process that links individuals with unique healthcare needs to services and resources in a synchronized effort to achieve optimal health outcomes [14]. Within HPTN 073, a theoretically grounded and culturally tailored intervention, herein referred to as client-centered care coordination or C4™, was used to support Black MSM to initiate and adhere to PrEP [15]. C4™ is a multi-level, multi-component intervention that is designed to be implemented in real-world clinical and community-based program settings. In HPTN 073, 178 Black MSM, who were provided C4, successfully initiated PrEP and adherence at 26 weeks was 64%. To scale-up PrEP among Black MSM using care coordination interventions, it is important to understand the time, costs, and personnel requirements for their effective implementation. The purpose of this paper is to describe the range of personnel, activities, costs, and time involved in the implementation of C4™ with PrEP-eligible Black MSM.

## Methods

The C4™ intervention was implemented in HIV Prevention Trials Network (HPTN) 073, a PrEP demonstration project among Black MSM (*N* = 226) in three cities including Chapel Hill, NC, Los Angeles, CA, and Washington, DC. A full description of the methods used in HPTN 073 is described in the primary outcome paper [15]. Participants in this non-randomized PrEP study were followed for 52 weeks, provided risk reduction counseling with C4, and offered PrEP. The study was approved by the institutional review boards of the University of California at Los Angeles, the University of North Carolina at Chapel Hill, and George Washington University. All participants provided informed consent.

**Fig. 1:**
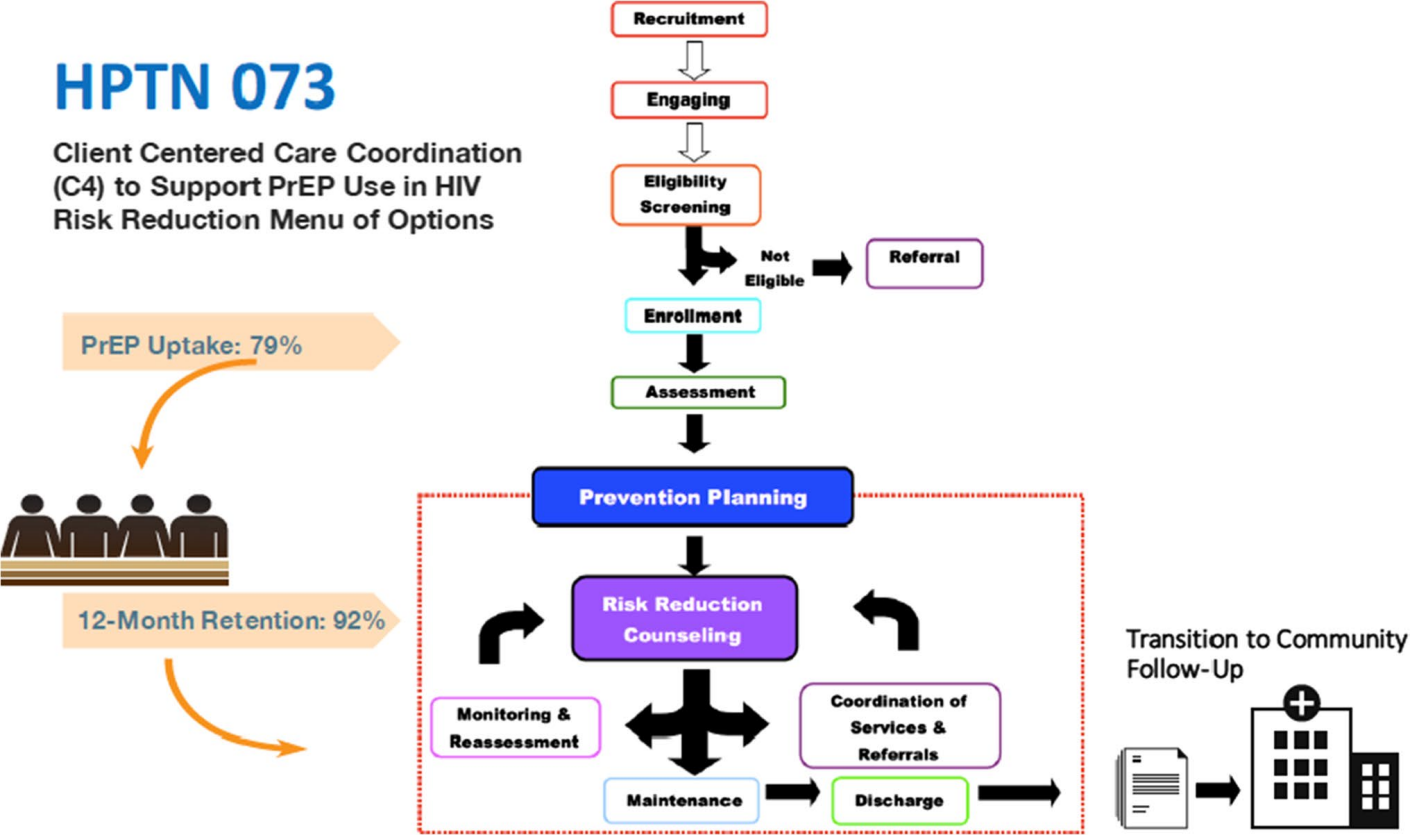
Client-centered care coordination process

C4™ consisted of prevention goal setting, intensive theory-based behavioral counseling, prevention action-plan development and monitoring, and per-participant as-needed care coordination. A case report form was used to record care coordination activities that occurred at study visits (both standard and ad hoc interim visits) as well as any care coordination that occurred between in-person scheduled study visits. Care coordination was not mandatory; thus, even if a care coordination-related need was identified either by the participant or the coordinator, care coordination could be declined by study participants at any time. The C4 process for the study is outlined in Fig. 1.

Study team members from each research site completed a three-day training on two main components of C4™. The first component of the training was a foundational grounding in self-determination theory (SDT) provided by Geoffrey C. Williams (University of Rochester Department of Medicine) and Christopher Niemic (University of Rochester, Department of Psychology)—two experts in SDT and its application to health promotion. SDT is a social-psychological theory of how social environments can be optimized to support human motivation for pro-health behaviors and is the organizing theoretical framework for C4™. Second, the University of Rochester Center for Community Practice—a CDC Part II Prevention Training Center at the time—provided training on Comprehensive Risk Counseling & Services (CRCS), which is a prevention case management program model in the CDC’s compendium of evidence-based interventions. The CRCS training included tailored content for how to apply the SDT concepts in the delivery of CRCS with Black MSM as well as skills-building on the use of CRCS strategies to support issues related to PrEP use and adherence. All participants were provided with a standardized C4™ implementation manual and access to on-demand technical assistance by the second author.

### Measures

#### Care Coordination

Care coordination activities were measured using an adapted version of the Care Coordination Measurement Tool (CCMT) [14]. The CCMT was used by personnel to note the activities performed to fulfill the care coordination needs of the participants they served. The tool includes items such as the focus of the encounters, total time spent with client (in minutes), the activities undertaken to fulfill clients’ needs (e.g., telephone discussions with client or service providers, electronic contact with clients or service providers, contact with client’s primary care provider (PCP), client medical chart review, and risk reduction goal chart plan revisions and/or updates), outcome of care coordination activities, and any follow-up or referrals needed (e.g., emergency department (ED) services received, community agencies, pharmacy or prescription needs, PCP follow-up, labs, or legal services). Site-based counselors who delivered C4 completed the CCMT after any activity that required

greater than 5 min of coordination of services. The tool was designed to ensure time spent on care coordination activities was documented to the nearest minute (see Fig. 1). The measure used in HPTN 073 was revised from the original CCMT to include items that increased its cultural and developmental relevance for adult Black MSM. Specifically, the tool was adapted for specific use on needs regarding PrEP to characterize the focus of the care coordination activities, the specific activities deployed, time expended, and outcomes achieved. Encounter outcomes were assessed by the provider conducting C4 sessions either by follow-up or perceived outcome.

#### Personnel Cost

Personnel costs were the costs associated with C4 components of the intervention. Personnel costs were calculated using the current median (mean) hourly rates for each occupation conducting C4 based on their metropolitan area using the US Bureau of Labor Statistics [16].

### Analysis

Statistical analysis consisted of descriptive statistical analysis: means and standard deviations, frequencies of items collected from the CCMT, and cost calculated using the US Department of Labor Statistics. All analysis was performed using the R software, version 3.4.1.

## Results

### Participant Characteristics

An approximately equal number of participants were recruited across the three cities: Los Angeles (*N* = 76), Chapel Hill (*N* = 75), and Washington DC (*N* = 75). Of those participants, 86.3% identified as African American, and 7.5%, 3.5%, and 0.4% identified as Afro-Latino, Afro-Caribbean, and African, respectively. During the study, 78.8% initiated PrEP; of those who initiated PrEP, 70.8% initiated within the first 30 days of study enrollment. One-third of participants (29.5%) were below the US poverty line with an annual income less than $10,000 [17]. Additionally, one-third of participants did not have health care coverage (31.4%) (Table 1).

**Table 1:** Demographic and site characteristics

	Total	Los Angeles	Chapel Hill/Durham	Washington DC
	(*N* = 226)	(*N* = 76)	(*N* = 75)	(*N* = 75)
Ethnicity: count (%)				
African American	195 (86.3)	62 (81.6)	70 (93.3)	63 (84.0)
African	1 (0.4)	1 (1.3)	0 (0.0)	0 (0.0)
Afro-Caribbean	8 (3.5)	1 (1.3)	1 (1.3)	6 (8.0)
Afro-Latino	17 (7.5)	9 (11.8)	4 (5.3)	4 (5.3)
Other	5 (2.2)	3 (3.9)	0 (0.0)	2 (2.7)
Annual income (thousands USD): count (%)				
Less than 5	48 (21.5)	24 (31.6)	17 (22.7)	7 (9.7)
5–10	20 (9.0)	9 (11.8)	7 (9.3)	4 (5.6)
1 –20	40 (17.9)	17 (22.4)	17 (22.7)	6 (8.3)
20–30	32 (14.3)	10 (13.2)	15 (20.0)	7 (9.7)
30–40	23 (10.3)	6 (7.9)	8 (10.7)	9 (12.5)
40–50	9 (4.0)	3 (3.9)	1 (1.3)	5 (6.9)
50–60	13 (5.8)	3 (3.9)	3 (4.0)	7 (9.7)
60–70	12 (5.4)	1 (1.3)	2 (2.7)	9 (12.5)
70–80	4 (1.8)	2 (2.6)	0 (0.0)	2 (2.8)
80 or more	22 (9.9)	1 (1.3)	5 (6.7)	16 (22.2)
Health care coverage available: count (%)				
Yes	155 (68.6)	48 (63.2)	47 (62.7)	60 (80.0)
No	71 (31.4)	28 (36.8)	28 (37.3)	15 (20.0)
Number of C4 encounters per participant				
Total	1135	377	212	546
Mean (SD)	5.0 (2.8)	5.0 (2.7)	2.8 (1.6)	7.3 (1.7)
Number of C4 encounters by staff role				
Counselor	1093 (96.3)	368 (97.6)	188 (88.7)	537 (98.4)
Nurse practitioner	20 (1.8)	1 (0.3)	18 (8.5)	1 (0.2)
Social worker	11 (1)	4 (1.1)	0 (0)	7 (1.3)
Registered nurse	5 (0.4)	0 (0)	5 (2.4)	0 (0)
Physician assistant	3 (0.3)	3 (0.8)	0 (0)	0 (0)
Family therapist	2 (0.2)	1 (0.3)	0 (0)	1 (0.2)
HIV tester	1 (0.1)	(0)	1 (0.5)	0 (0)

### Care Coordination Encounters

During the study, the total number of care coordination encounters was 1,135, with an average of 5.0 encounters per participant. The highest proportion (48.1%) of encounters occurred in Washington DC (*n* = 546), followed by Los Angeles 33.2% (*n* = 377) and Chapel Hill 18.7% (*n* = 212) sites. The care coordination encounters covered a range of health and social support topics. Table 2 displays the distribution of care coordination encounter foci across the three cities. The most common focus of these encounters was PrEP adherence support (*n* = 492, 43.3%), sexual health services (*n* = 217, 19.1%), or social services (*n* = 168, 14.8%) such as housing, food, and clothing.

**Table 2:** Focus of care coordination encounter

Primary focus	Total(*N* = 1135)	Los Angeles(*n* = 377)	Chapel Hill/Durham (*n* = 212)	Washington DC (*n* = 546)
PrEP adherence support	492 (43.3)	178 (47.2)	65 (30.7)	249 (45.6)
Clinical and medical management	67 (5.9)	21 (5.6)	38 (17.9)	8 (1.5)
Referral management	39 (3.4)	12 (3.2)	19 (9.0)	8 (1.5)
Social services^1^	168 (14.8)	43 (11.4)	21 (9.9)	104 (19.0)
Sexual health services	217 (19.1)	76 (20.2)	35 (16.5)	106 (19.4)
Mental health	52 (4.6)	20 (5.3)	22 (10.4)	10 (1.8)
Legal/judicial	3 (0.3)	1 (0.3)	2 (0.9)	0 (0.0)
Substance use treatment	47 (4.1)	18 (4.8)	6 (2.8)	23 (4.2)
Employment	40 (3.5)	2 (0.5)	1 (0.5)	37 (6.8)
Linkage to care for HIV infection	10 (0.9)	6 (1.6)	3 (1.4)	1 (0.2)

^1^Social services refers to such items as housing, food, and clothing

Care coordination activities were primarily conducted by counselors (96.3%), followed by nurse practitioners (1.8%) and social workers (1.0%). Other healthcare workers who performed care coordination were registered nurses, physician assistants, family therapists, and HIV risk reduction counselors; however, each of these roles accounted for < 1% of care coordination encounters. The time that health care workers spent on care coordination activities to address the needs of their clients varied from 5 min per encounter to > 50 min, with most encounters taking between 30 and 39 min.

A total of 1,331 recorded activities occurred over the 1,135 encounters. The most frequently performed activities were as follows: developing or modifying an existing written prevention plan (*n* = 383; 28.8%), chart review (*n* = 251; 18.9%), and telephone discussions directly with the participant being served (*n* = 164; 12.3%). The activities performed to fulfill care coordination needs varied across sites. In Los Angeles, developing or modifying an existing care plan (*n* = 264; 34.8%) was the most common activity performed. In Washington DC, developing and modifying an existing written prevention plan (*n* = 118; 51.5%) was also most performed. Chapel Hill differed from both sites, with telephone communication directly with participants (*n* = 131; 38.2%) and client-focused research (*n* = 64; 18.7%) were recorded as the most common activities performed to fulfill the participant’s care coordination needs. A full description of the activities performed at each site is listed in Table 3.

**Table 3:** Client-centered care coordination activities and outcomes

	Total	Los Angeles	Chapel Hill/Durham	Washington DC
Number of encounters	1135	377	212	546
Activities (%)				
Telephone discussion				
Client	164 (12.3)	26 (3.4)	131 (38.2)	7 (3.1)
Family	3 (0.2)	–	2 (0.6)	1 (0.4)
Agency	66 (5.0)	19 (2.5)	34 (9.9)	13 (5.7)
Hospital/clinic	61 (4.6)	16 (2.1)	44 (12.8)	1 (0.4)
Pharmacy	1 (0.1)	–	1 (0.3)	–
Electronic message				
Client	39 (2.9)	8 (1.1)	21 (6.1)	10 (4.4)
Agency	21 (1.6)	1 (0.1)	6 (1.7)	14 (6.1)
Hospital/clinic	16 (1.2)	–	14 (4.1)	2 (0.9)
Contact with consultant				
Telephone	3 (0.2)	3 (0.4)	–	–
Meeting	9 (0.7)	6 (0.8)	3 (0.9)	–
Forms processing	2 (0.2)	–	2 (0.6)	–
Confer with primary care physician	1 (0.1)	–	1 (0.3)	–
Chart review	251 (18.9)	242 (31.9)	5 (1.5)	4 (1.7)
Develop/modify written care plan	383 (28.8)	264 (34.8)	1 (0.3)	118 (51.5)
Meeting/case conference	73 (5.5)	41 (5.4)	11 (3.2)	21 (9.2)
Client-focused research	228 (17.1)	127 (16.7)	64 (18.7)	37 (16.2)
Number of outcomes occurred (%)	1700	808 (47.5)	294 (17.3)	598 (35.2)
Advised family/client on home management	3	–	–	3 (0.5)
Referral to specialist	33 (1.9)	8 (1.0)	11 (3.7)	14 (2.3)
Referral for hospitalization	–	–	–	–
Referral for primary care office visit	86 (5.1)	51 (6.3)	29 (9.9)	6 (1.0)
Referral to lab/X-ray	14 (0.8)	–	14 (4.8)	–
Referral to community agency	158 (9.3)	74 (9.2)	66 (22.4)	18 (3.0)
Referral to specialized therapies	17 (1.0)	13 (1.6)	3 (1.0)	1 (0.2)
Ordered prescription, equipment, taxi, etc	186 (10.9)	184 (22.8)	2 (0.7)	–
Reconciled discrepancies (inc. missing data, miscommunication, compliance issues, etc.)	10 (0.6)	–	8 (2.7)	2 (0.3)
Reviewed labs, specialist reports, etc	133 (7.8)	119 (14.7)	7 (2.4)	7 (1.2)
Advocacy for family/client	34 (2.0)	28 (3.5)	–	6 (1.0)
Met client’s immediate needs, questions, and concerns	1011 (59.5)	326 (40.3)	144 (49.0)	541 (90.5)
Outcome pending	15 (0.9)	5 (0.6)	10 (3.4)	–

### Care Coordination Outcomes

During the entire study, the 1,135 C4™ encounters generated 1,700 encounter outcomes across the three study sites. Overall, personnel reported that 59.5% of those encounters met their participants’ immediate needs, questions, and concerns; however, these reports were distributed disproportionately across the three sites, with 90.5%, 49.0%, and 40.3% from Washington DC, Chapel Hill, and Los Angeles, respectively. In Chapel Hill and Washington DC, the second most frequent outcome resulting from a care coordination encounter was referral to a community agency, while in Los Angeles it was instrumental support (i.e., ordering prescription medications, equipment, taxi, etc.), which was followed by referral to a community agency. Other referrals included referral to a primary care office, lab, or specialist (Table 3).

### Care Coordination Personnel Costs

A total of 549.6 personnel hours of C4 intervention delivery were logged related to 1,135 C4 encounters. The total personnel costs of care coordination were $9,826, resulting in a mean cost of $8.70 per client encounter. The average time for each encounter was 29 min, which varied by personnel type (range: 17–40 min). The lowest cost per encounter was with an HIV tester ($7.10/encounter) and the highest was with a registered nurse ($21.30/encounter). Most encounters occurred with counselors (*n* = 1,093, 96.3%), resulting in 531.8 personnel hours. A full breakdown of care coordination activities by personnel type and implementation city can be found in Table 4.

**Table 4:** Cost of client-centered care coordination by personnel type

Personnel type	Encounters	Total time spent	Mean time enctr	Total cost	Mean cost enctr
	Count (%)	Hours (%)	Min	USD (%)	USD
Total	1135	549.6	29^a^	9826^b^	8.7^c^
Counselor	1093 (96.3)	531.8 (96.8)	29	9118 (92.8)	8.3
Nurse practitioner	20 (1.8)	5.7 (1.0)	17	319 (3.3)	16.0
Social worker	11 (1.0)	6.7 (1.2)	36	211 (2.1)	19.2
Registered nurse	5 (0.4)	3.3 (0.6)	40	107 (1.1)	21.3
Physician assistant	3 (0.3)	1.0 (0.2)	20	49 (0.5)	16.3
Family therapist	2 (0.2)	0.6 (0.1)	18	15 (0.2)	7.5
Tester	1 (0.1)	0.5 (0.1)	30	7 (0.1)	7.1

^a^Mean time per encounter varied by site: Los Angeles (35 min), Chapel Hill/Durham (24 min), Washington DC (27 min)

^b^Total cost (USD) varied by site: Los Angeles (3,793), Chapel Hill/Durham (1,940), Washington DC (4,093)

^c^Mean cost per encounter (USD) varied by site: Los Angeles (10.1), Chapel Hill/Durham (9.2), Washington DC (7.5)

## Discussion

In this paper, we described the activities, personnel costs, and encounter outcomes of the implementation of novel theory-based care coordination intervention implemented to support HIV prevention—including PrEP use—for Black MSM in three US cities. Men enrolled in the study received coordinated care services in which the case worker for the client either provided services directly to the client (e.g., HIV testing, PrEP adherence counseling) or made active referrals to other community agencies for services not offered by the organization (e.g., mental health services, legal services, housing services). The case workers followed up with clients to assess their access of the services and provide additional support for the services accessed, if needed. As self-determination was an essential philosophy of the intervention, the social determinants of health addressed by in intervention staff we driven by the needs identified by participants. Sexual health and social services, including PrEP adherence counseling, were the most frequent focus of care coordination encounters. Participants also accessed care coordination for mental health, medical services, and substance use treatment. The range of services used by participants illustrates the breadth of service needs for Black MSM. These findings highlight the need for the availability and accessibility of services that can support Black MSM’s psychosocial needs and address the negative impacts of clinical, psychosocial, and structural factors on their engagement in HIV prevention services. These findings supports existing research which argues that to reduce HIV incidence in Black MSM, HIV prevention interventions must address social determinants of health [18, 19]. Furthermore, these findings reaffirm that addressing comorbidities facilitates successful engagement in HIV prevention activities, including PrEP use for Black MSM [20–23].

The broad service encounters resulted in a large portion of time being dedicated to development and modifying care plans and the coordinator researching services to meet the immediate needs of the HPTN 073 participants. These activities suggest that implementation of C4™ requires ongoing knowledge of community supports and services, maintaining relationships with community-based organizations and social service providers, and resources available to support clients in accessing support and auxiliary services (e.g., transportation funding, emergency funds). In HPTN 073, the ability to facilitate services was dependent upon availability of services in the community; thus, implementation of C4™ is highly impacted by range of services in the community. Higher resourced communities may be able to facilitate greater access to service needs identified by clients. These findings are similar to research which suggest the availability of community services influences health outcomes, with greater resourced communities having better health outcomes [24–26].

There are numerous costs involved with leveraging a C4 program that are embedded within the research effort and would need to be considered when launching a program in a real-world setting. Particularly in network studies with existing research infrastructure already established, the costs of implementation may seem deceptively small because key program functions are already paid for and not counted within the specific study budget. Specific potential implementation costs are outlined in Table 5. In this paper, we describe the cost of C4 as it relates to personnel costs. C4™ personnel costs were relatively low cost. The majority (96%) of care coordination encounters were conducted by counselors. The bulk of the other service encounters (2.8%) were completed by social workers and nurse practitioners which suggests that client-centered care coordination can be implemented in settings without heavy utilization of licensed healthcare professional staff, incurring lower salary expenses. One of the challenges to implementing HIV prevention interventions in the USA and Canada is the lack of HIV prevention resources in rural counties [27–30]. Due to the low cost associated with staffing, C4™ has the potential to be an effective and low-cost way to provide care coordination services in rural and other resource constrained settings. Specifically, rural service providers maybe able to implement aspects of C4 into their existing practice to assist Black MSM access support services to complement engagement in HIV prevention services. This has an important practical implication given the identification of rural areas in the Ending the HIV Epidemic initiative [31] as key areas for HIV prevention prioritization. This includes several states in the US south (i.e., Alabama, Arkansas, Kentucky, Mississippi, and South Carolina) where Black MSM have disproportionately high HIV incidence and prevalence. One challenge which needs to be addressed in rural communities is the lack of service providers, particularly service providers who are cultural responsive to Black MSM [19, 21]. C4 does not address the gap in access to health services; however, it has the potential to address the need for services being co-located to reduce barriers of access. Within the care coordination setting, nursing and social work professionals can be optimized by utilizing services that fall within their scope of practice and not assigning them additional responsibilities that can be performed by other members of the team, maintaining lower cost for licensed professionals. This can be especially useful when considering the increase in HIV expenditures due to the rise in comorbid conditions resulting from HIV, such as cardiovascular diseases and chronic kidney disease [32].

**Table 5:** Program cost and resources considerations

Total program cost considerations	
Space for consultations	Resources to establish and maintain community partnerships
Telecommunications (internet, phones, computer, mobile devices)	Community engagement strategies including a CAB
Intervention training	Methods for documentation of services delivered
Transportation for staff and clients	Program monitoring and evaluation of program
Ongoing training and continuing education	Staff supervision

Implementation of C4 in this PrEP demonstration study proved to address the holistic needs of Black MSM; however, this intervention is not without limitations. Because C4 was embedded in a network study, the total costs of implementation (e.g., infrastructure) costs are not accounted for in this study. However, this study was able to parse out specifically the C4 delivery costs from the overall program costs. While that does not give much in terms of being able to figure out overall program costs, it does have the unique benefit of being generalizable since it looks at minutes of delivery. If a local organization wanted to implement C4, they could look at the specific time commitment/resources needed to add C4 specifically onto their existing program offerings. While personnel costs were relatively low, the expenditures at each site varied based on the differences in salaries and the availability of resources. Furthermore, the study was not designed to evaluate the cost-effectiveness of the intervention; therefore, implementation of this intervention would need to account for indirect costs associated with implementation of C4. Future studies of C4 should determine full intervention costs to determine cost-effectiveness. Personnel mean hourly wage used in C4™ varied by personnel type and location, but it was consistently highest for nurse practitioners (*M* = $56.08), followed by physician assistants (*M* = $47.21) and registered nurses (*M* = $38.27). The average mean hourly wage across personnel type was highest in Los Angeles ($35.65), and similar within Washington DC ($32.49) and Chapel Hill ($31.50). These costs were based on mean personnel costs based on the US Bureau of Labor Statistics and actual costs were not assessed in the study. The implementation of C4™ may be highly variable based on the availability of community resources and labor costs of the available healthcare facility staff. Our study findings suggest that care coordination outcomes were more advantageous in Washington DC site than in Chapel Hill and Los Angeles. Unfortunately, there was no qualitative follow-up with clients to determine why care coordination was more advantageous in certain cities. Further research is needed to determine the impact of community-level factors on the engagement of C4 and how availability of community resources might impact the overall outcome of engagement in C4. It might be due to participant resources outside of the intervention impacted their engagement in C4. Research demonstrates that those who have more resources are more likely to benefit from HIV interventions [21, 33].

## Conclusions

A client-centered care coordination intervention—C4™—was successfully implemented across three cities in the HPTN 073 study. Although client-centered care coordination has been implemented in well-resourced communities with robust HIV research and service infrastructure, our findings suggest that C4™ can also be successfully implemented in lower resourced communities. C4™ program activities can be performed by most staff at organizations, reducing the provider associated costs and burden. Understanding the activities, personnel, cost, and time needed for successful implementation of care coordination interventions is critical administrative evidence for informing future programmatic dissemination, implementation, and stainability.

## Data Availability

Not applicable.
